# Characterization of the fecal and mucosa-associated microbiota in dogs with colorectal epithelial tumors

**DOI:** 10.1371/journal.pone.0198342

**Published:** 2018-05-31

**Authors:** Kristin Marie Valand Herstad, Aina Elisabeth Fossum Moen, John Christian Gaby, Lars Moe, Ellen Skancke

**Affiliations:** 1 Department of Companion Animal Clinical Sciences, Faculty of Veterinary Medicine, Norwegian University of Life Sciences (NMBU), Oslo, Norway; 2 Department of Clinical Molecular Biology (EpiGen), Akershus University Hospital, Lørenskog and University of Oslo, Oslo, Norway; 3 Department of Chemistry, Biotechnology and Food Science, Norwegian University of Life Sciences (NMBU), Ås, Norway; "INSERM", FRANCE

## Abstract

Colorectal epithelial tumors occur spontaneously in dogs, and the pathogenesis seems to parallel that of humans. The development of human colorectal tumorigenesis has been linked to alterations in the composition of the intestinal microbiota. This study characterized the fecal- and mucosa-associated microbiota in dogs with colorectal epithelial tumors (n = 10). The fecal microbiota was characterized by 16S rDNA analysis and compared with that of control dogs (n = 13). We also determined the mucosa-associated microbiota composition in colonic tumor tissue (n = 8) and in adjacent non-tumor tissue (n = 5) by 16S rDNA- and rRNA profiling. The fecal microbial community structure in dogs with tumors was different from that of control samples and was distinguished by oligotypes affiliated with *Enterobacteriaceae*, *Bacteroides*, *Helicobacter*, *Porphyromonas*, *Peptostreptococcus* and *Streptococcus*, and lower abundance of *Ruminococcaceae*, *Slackia*, *Clostridium* XI and *Faecalibacterium*. The overall community structure and populations of mucosal bacteria were not different based on either the 16S rDNA or the 16S rRNA profile in tumor tissue *vs*. adjacent non-tumor tissue. However, the proportion of live, potentially active bacteria appeared to be higher in non-tumor tissue compared with tumor tissue and included *Slackia*, *Roseburia*, unclass. *Ruminococcaeceae*, unclass. *Lachnospiraceae* and *Oscillibacter*. Colorectal tumors are rarely diagnosed in dogs, but despite this limitation, we were able to show that dogs with colorectal tumors have distinct fecal microbiota profiles. These initial results support the need for future case-control studies that are adequately powered, as well as age-matched and breed-matched, in order to evaluate the influence of bacteria on colorectal cancer etiopathogenesis and to determine whether the bacteria may have potential as biomarkers in clinical settings.

## Introduction

In dogs colorectal epithelial tumors occur spontaneously, and similarly to humans, adenocarcinoma is one of the most common malignant tumors. Sporadic colorectal adenocarcinoma in humans often arises from benign polyps that develop into adenomas, and it involves multiple steps of genetic and epigenetic alterations [1]. This same developmental process is also thought to occur in dogs [2–5]. In humans, genetic predisposition, diet, environment and intestinal bacteria are implicated in the etiopathogenesis [6–10]. Intestinal bacteria with pro-carcinogenic properties, such as *Helicobacter pylori*, *Escherichia coli*, *Streptococcus gallolyticus* (formerly *bovis*), *Fusobacterium* spp., and *Bacteroides fragiles* have been identified in fecal or tumor samples from human patients with adenoma and carcinoma [11–15]. Presence of potentially pathogenic bacteria and/or bacterial dysbiosis is commonly observed in these patients [16, 17]. Current evidence suggests that rather than only one pathogenic microbe, a complex network of microbes is involved in the pathogenesis of disease [17, 18].

In dogs, bacterial dysbiosis has been described in association with acute diarrhea and inflammatory bowel disease (IBD) [19–21]. One study reported changes in the intestinal microbiota of dogs with colonic enteropathies, including colorectal adenocarcinomas (n = 9) and lymphosarcoma (n = 3), but only select bacterial populations were characterized [22–24]. Whether dysbiosis is evident in dogs with colorectal epithelial tumors, based on methods evaluating the entire communities of bacteria, is currently unknown.

Studies in microbial ecology commonly use the 16S small subunit ribosomal DNA (rDNA) as a taxonomic marker gene to characterize bacterial populations because this gene is universally conserved among prokaryotes. The 16S rDNA data provides a snapshot of all bacteria present regardless of whether they are metabolically active, dormant or dead. Sequence data derived from 16S rRNA serves as an indicator of metabolically active bacteria since actively dividing bacterial cells generally express higher amounts of rRNA than dormant or dead bacteria [25].

The characterization of microbes in the distal part of the colon and rectum is commonly accomplished by collecting fecal samples because it is non-invasive. Distinct fecal microbial communities were detected in human patients with early *vs*. late stages of cancer, providing evidence that microbiota could serve as biomarkers in order to aid in the diagnosis and management of human colorectal cancer [17]. Despite wide use, fecal samples may contain transient organisms that may not reflect the mucosa-associated microbiota [26]. Hence, it may be more relevant to characterize and compare the mucosa-associated microbiota in tumor tissue with that of non-tumor tissue, so as to identify bacteria potentially involved in tumorigenesis [27, 28].

The lack of knowledge as to whether the intestinal microbiota changes with the development of colorectal epithelial tumors in dogs prompted us to (1) compare the fecal microbiota of dogs with colorectal tumors to that of control dogs and to (2) compare the mucosa-associated microbiota in tumor tissue with that of adjacent non-tumor tissue. For these purposes, we used high throughput sequencing (HTS) methods to obtain amplicons from rDNA and rRNA. We identified differentially abundant fecal bacterial taxa in dogs with tumors *vs*. control dogs—taxa which could be involved in the pathogenesis of colorectal epithelial tumors and could serve as biomarkers in clinical settings for diagnostic, prognostic, and therapeutic purposes.

## Materials and methods

The study protocol was reviewed and approved according to the guidelines of the ethics committee at the Faculty of Veterinary Medicine, Norwegian University of Life Sciences (NMBU) (approval number: 14/04723). Written informed consent was given by all dog-owners before participation, and they were informed that their participation in the study was voluntary.

### Animals

#### Dogs with colorectal tumors

Client-owned dogs (n = 10) diagnosed with colorectal epithelial tumors were recruited to a prospective case study over a two-year period. An overview of the demographics of the cohort and the samples used for analysis are shown in Table 1. The dogs consisted of various breeds and genders. Their age ranged from 2 to 14, with a median age of 9 years. Diets consisted of various types of dry food (Table 2). The tumors were in the distal part of the gastrointestinal tract, located within 10 cm proximal from the anus. Histopathological diagnosis included polyps (n = 2), adenomas (n = 5), and carcinomas (n = 3). Histopathology was evaluated by a board-certified veterinary pathologist according to the guidelines developed by the World Small Animal Veterinary Association and was based on the WHO International Histological Classification of Tumors in Domestic Animals [29].

**Table 1:** Overview of dogs and samples included in the study.

Dog id^1^	Breed	Age	Bw^2^ (kg)	Sex^3^	Examination	Tumor mucosa	Adjacent non-tumor tissue	Fecal sample	Histopathology
***Dogs with tumors***									
1	Mixed breed	4	UN	M	Surgery	yes	no	yes	Polyp
2	Golden Retriever	5	UN	F	Surgery	no	no	yes	Polyp
3	Havanese	5	7	F	Colonoscopy	yes	yes	no	Adenoma
4	Golden Retriever	2	38	M	Surgery	yes	no	yes	Adenoma
5	Gordon Setter	10	21	F	Surgery	yes	no	yes	Adenoma
6	English Springer Spaniel	8	24	M	Colonoscopy	yes	yes	yes	Adenoma
7	English Setter	10	23	FN	Colonoscopy	yes	yes	no	Adenoma
8	Mixed breed	10	9	MN	Necropsy	yes	yes	yes	Adenocarcinoma
9	Shetland Sheepdog	14	11	M	Necropsy	no	no	yes	Adenocarcinoma
10	Am. Cocker Spaniel	10	12	F	Colonoscopy	yes	yes	yes	Adenocarcinoma
***Control dogs***^***4***^^,^^***5***^									
11	Coton de Tulear	9	7	F	Necropsy	NA	NA	yes	Normal colon
12	Rottweiler	4	50	M	Necropsy	NA	NA	yes	Normal colon
13	Irish Setter	10	15	F	Necropsy	NA	NA	yes	Normal colon
14	English Springer Spaniel	8	20	F	NA	NA	NA	yes	NA
15	Mixed breed	3	15	F	NA	NA	NA	yes	NA
16	Small Munsterlander	6	22	F	NA	NA	NA	Yes	NA
17	Irish Setter	4	22	M	NA	NA	NA	Yes	NA
18	Mixed breed	5	15	M	NA	NA	NA	Yes	NA
19	English Setter	5	25	M	NA	NA	NA	Yes	NA
20	English Cocker Spaniel	3	19	M	NA	NA	NA	Yes	NA
21	Mixed breed	6	29	F	NA	NA	NA	Yes	NA
22	English Cocker Spaniel	8	10	F	NA	NA	NA	Yes	NA
23	German Shorthaired Pointer	3	20	F	NA	NA	NA	Yes	NA

^1^Dog identifier (id) number

^2^ UN: unknown

^3^F:female; M:male; N:neutered

^4^No.14 to 23 Participated in a previously performed dietary intervention study [30]

^5^NA: not applicable

**Table 2:** Overview of diets given to dogs in this study.

Dog id^1^	Diet^2^
Dogs with tumors	
1	Royal Canin Adult dry food
2	Purina Proplan dry food
3	Royal Canin Adult Yorkshire terrier dry food, Hill`s Prescription Diet i/d dry food, various types of canned food and table scrapes
4	Eukanuba Adult dry food
5	Royal Canin Adult 7+ dry food
6	Royal Canin Sensible dry food, Hill`s Prescription Diet j/d dry food, Hill`s Prescription Diet a/d canned food.
7	Royal Canin setter dry food
8	Eukanuba Dermatosis dry food
9	UN
10	UN
Control dogs	
11	UN
12	Hill`s Prescription Diet j/d dry food
13	UN
14	Felleskjøpet Labb Adult dryfood
15	Felleskjøpet Labb Adult dryfood
16	Felleskjøpet Labb Adult dryfood
17	Felleskjøpet Labb Adult dryfood
18	Felleskjøpet Labb Adult dryfood
19	Felleskjøpet Labb Adult dryfood
20	Felleskjøpet Labb Adult dryfood
21	Felleskjøpet Labb Adult dryfood
22	Felleskjøpet Labb Adult dryfood
23	Felleskjøpet Labb Adult dryfood

^1^Dog identifier (id) number

^2^UN: unknown

None of the dogs had any history of inflammatory bowel disease or any other gastrointestinal disease, and no antibiotic treatments had been given during the last three months prior to sample collection.

#### Control dogs

The control dogs (n = 13) consisted of various breeds and genders, and their age ranged from 3 to 10 with a median age of 5 years. Ten of these dogs (dog nos.14-23, Table 1) had participated in a previously performed prospective dietary intervention study at NMBU [30]. These ten dogs had consumed similar dry food (Labb Adult, Felleskjøpet, Norway) for two weeks prior to sample collection. Prior to that, they had received various types of dry food (S1 File). The remaining three dogs (dog nos.11-13, Table 1) were included during the study period. They were euthanized due to non-gastrointestinal disorders related to aggressive behavior in two dogs, and dystocia in the third. The detailed demographics of the dogs are described in Table 1 and in the previous study [30]. In order to be included, dogs had to be clinically healthy, and no treatment with antibiotics was given within the last six months prior to sample collection.

### Samples

#### Fecal samples

Fecal samples were collected from 10 dogs diagnosed with colorectal epithelial tumors and from 13 healthy dogs that comprised the control group. For ten control dogs (14–23, Table 1), samples were taken after the first dry food period (CD1) in the dietary intervention study described in [30]. The owners were instructed to collect one fecal sample from their dog immediately after natural defecation, thereby limiting contamination from the ground as much as possible. In order to avoid biased fecal microbiota composition, samples were obtained prior to fasting and bowel cleansing procedures. Where post mortem examinations were performed, feces was obtained directly from the rectal lumen immediately after euthanasia. Each sample was put in hygienic sample vials as supplied by the investigator. The samples were either frozen within one hour in the owner’s home freezer and then transported on ice to the laboratory for storage at -80°C, or immediately frozen at -80°C during necropsy.

#### Tissue samples

Eight of ten dogs contributed colonic mucosal tissue from tumor collected by colonoscopy (n = 4), surgical excision (n = 3) or necropsy (n = 1). Adjacent non-tumor tissue was collected from dogs through colonoscopy and necropsy, which encompassed five of the eight dogs (Table 1). Non-tumor tissue was not obtained from dogs where tumors were removed through surgery for ethical reasons. Non-tumor tissue was obtained about 10 cm proximal to the tumor. The samples were collected by biopsy forceps during colonoscopy, and through mucosal incision when retrieved by surgical excision or necropsy. Prior to colonoscopy and surgical removal of tumors, dogs fasted for 48 hours and bowel cleansing was performed using Laxabon (BioPhausia, Stockholm, Sweden) at 30 ml/kg orally. An additional rectal cleansing step using 20 ml/kg warm water was performed during anesthesia immediately prior to the colonoscopy.

From three of the control dogs, healthy colonic mucosal samples were collected immediately after euthanasia. No abnormalities were revealed during histopathological examination of colonic mucosal tissue from these dogs and of non-tumor tissue from tumorous dogs.

Colonic tissue samples were fixed in 10% neutral buffered formalin, embedded in paraffin, sectioned, and stained with hematoxylin and eosin for histopathological interpretation. Additional samples were placed in Allprotect Tissue Reagent (Qiagen, Hilden, Germany) immediately after collection and stored according to the manufacturer’s instructions.

### Isolation of DNA from fecal samples

Fecal samples were thawed on ice and ~ 200 mg from each sample was added to sterile water at a ratio of 1:3. Homogenization involved bead beating using a MagNaLyser (Roche, Basel, Switzerland) twice at 6500 rpm for 20 s with 1 minute cooling at 4°C between runs as described previously [30]. DNA was extracted using the Mag Mini LGC kit (LGC Genomics, Hoddesdon, UK) according to the manufacturer's recommendations using a KingFisher Flex DNA extraction robot (Thermo Fisher Scientific, Waltham, MA, USA). Adequate DNA quality and quantity in samples were ensured using a NanoDrop ND-1000 spectrophotometer (Thermo Fisher Scientific). DNA samples were stored at -20°C until processing.

### Isolation of DNA and RNA from mucosal samples and cDNA synthesis

Using the AllPrep DNA/RNA Mini Kit (Qiagen), RNA and DNA were isolated from ~ 8 mg of mucosal tissue that had been preserved in Allprotect Tissue Reagent (Qiagen). The manufacturer’s instructions were followed except for extended homogenization and additional enzymatic lysis steps as reported in [31]. For optimal RNA purification, on column DNAse treatment was included as described in the DNA/RNA Mini Kit protocol. RNA and DNA were eluted with 40 μl nuclease free water (NFW) and stored at −80°C and −20°C, respectively. The RNA and DNA concentrations were assessed using NanoDrop ND-1000 spectrophotometer (Thermo Fisher Scientific). For RNA quality the RNA integrity number (RIN) was tested using the Agilent 2100 Bioanalyzer (Agilent Technologies Inc., Santa Clara, CA, USA), the Agilent 2100 Expert Software and the Agilent RNA 6000 Nano Kit. cDNA was synthesized from 200 ng RNA using the AccuScript High Fidelity 1st Strand cDNA Synthesis Kit (Agilent Technologies Inc.) with random hexamers according to the manufacturer’s instructions. Two RNA samples were run in the absence of reverse transcriptase to assess the degree of contaminating genomic DNA. To verify synthesis of microbial cDNA, a real-time PCR amplification was performed using universal primers targeting the 16S rRNA [32] and was run on the ABI Prism 7900HT Real Time PCR System running the software SDS 2.4 (Thermo Fisher Scientific). [32]The PCR amplifications were performed in triplicate using a final reaction volume of 20 μl with 10 μl Power SYBR Green PCR Master mix (Thermo Fisher Scientific), 4 μl of 5 μM primer mix, 2 μl cDNA and 4 μl nuclease-free water using default cycling conditions. cDNA was stored at -20°C until further processing.

PCR amplification of the hypervariable region V4 of the 16S rRNA gene was performed following the Patric Schloss lab protocol “Miseq Wet Lab SOP” [33, 34], using the pad-linker-gene primers described therein, but applying some modifications of the template concentrations [33, 34]. The V4 region was selected since we aimed for full overlap of the 250 base reads, as this approach reduces the risk of sequencing errors [34]. The nucleotide sequences for the indexed primers used in the present study are listed in S2 File. The final PCR reaction concentrations consisted of 1 μM of each primer plus 25 ng/μl template for mucosal DNA and 0.9 μM of each primer plus 87 ng/μl template for both the fecal DNA and mucosal cDNA. Both reactions contained 17 μl AccuPrime^TM^ Pfx Supermix (Agilent Technologies Inc.). For fecal DNA and mucosal cDNA, 4 μl of template was added, whereas for mucosal DNA, 1 μl of 500 ng/μl was added, resulting in a final volume of either 20 or 23 μl. The PCR cycling conditions were 95°C for 2 min followed by 30 cycles of 95°C for 20 s, 55°C for 15 s and 72°C for 5 s, and then a final step of 72°C for 5 min. The PCR products were then stored at 4°C. Gel electrophoresis using 1% agarose gel confirmed the expected amplicon size (~ 400 bp) for all samples. A total of 3 μl of each amplicon was added to one of three pools separated according to the intensity of gel bands (classified as weak, moderate or strong). The pooled samples were run on a 3% agarose gel in 1xTAE at 60 V for one hour. Each band was carefully excised from the gel and nucleic acids were extracted using QIAquick Gel Extraction Kit (Qiagen), according to manufacturer’s instructions. Quantification of the pooled libraries was performed using the KAPA Library Quantification Kit Illumina® Platforms (Kapa Biosystems, Wilmington, MA, USA), following the manufacturer’s instructions. The three pools were finally combined according to concentrations and number of samples in each pool. The final combined library was diluted to 4 nM and sequenced using the MiSeq sequencing platform (Illumina Inc., San Diego, CA, USA) and the 500 cycle MiSeq Reagent Kit v2 with addition of custom sequencing primers, index and 10% phiX, as described in the “Miseq Wet Lab SOP” [33]. The MiSeq sequencing platform (Illumina) was hosted at the Department of Clinical Molecular Biology (Akershus University Hospital, Lørenskog, Norway).

### Sequence analysis

Mothur v.1.37.4 [35] was used to process the sequence data according to the protocol described in “MiSeq SOP” [34, 36]. Sequences were aligned with the Silva 16S rRNA reference database release 123. Any sequences not consistent with the target amplicon size (250 bp), containing any ambiguous base calls or homopolymers >8 bp, or that did not align properly were discarded. Chimeras were detected using the quality filtering pipeline UCHIME [37] and removed. The reads were subsequently clustered at 97% similarity into Operational Taxonomic Units (OTUs). Sequences were assigned taxonomy according to the RDP database with an 80% confidence threshold [38, 39]. The abbreviation “unclass.” corresponds to unclassified taxonomy within the respective taxonomic group. Samples were rarefied to 5500 sequences per sample before alpha and beta diversity analysis. The weighted UniFrac distance metric from mothur was used as input file to PRIMER 7 [40] to generate a 2-dimensional non-metric multidimensional scaling (nMDS) plot. Two-dimensional NMDS ordination of Bray-Curtis and binary Jaccard distances was accomplished with the R phyloseq [41], ggplot2 [42], cowplot [43], and vegan packages [44] within R software [45]. The rarefaction curve for observed OTUs was generated using QIIME [46]. Minimum Entropy Decomposition (MED) was used to separate between closely related taxa [47]. MED is a clustering independent approach that is sensitive to variation in the microbial community at the strain level. Raw FASTA sequences were merged using PEAR version 0.9.6 with a minimum overlap of 200 bp and an assembly length of 150–350 bp. Sequences were quality filtered using PRINSEQ lite version 0.20.4 with a min. length 150 bp, max. length 350 bp, min. quality score 20, and min. quality score mean 30. Short sequences were padded with gap characters before MED was performed. A representative sequence from each of the MED nodes was used as a query for the RDP database, with confidence threshold set to 80% [38]. To produce plots of of the differentially abundant oligotypes, an R phyloseq [41] object was made from the oligotype abundances and the metadata.

### Statistical analysis

Data were tested for normality using the Shapiro-Wilk normality test. Non-parametric Mann-Whitney U test was used to assess whether age, weight and gender were significantly different between dogs with tumors and control dogs (Prism7, GraphPad Software Inc, San Diego, CA). Estimators of population diversity (inverse Simpson’s index) and evenness (non-parametric Shannon’s evenness index) were compared between the clinical groups using the Mann-Whitney U test for non-paired data and the Wilcoxon matched-pairs signed rank test for paired data (Prism7, GraphPad Software Inc, San Diego, CA).

We normalized RNA (reflective of the live, potentially active bacteria) by DNA (reflective of the total number of bacteria) by calculating the RNA/DNA ratio for each OTU at genus level in each sample. OTUs with RNA/DNA ratio of 0 were removed. We plotted an XY scatterplot of the median values of RNA/DNA ratios of OTUs in tumor and non-tumor tissue using Excel 2013. Wilcoxon signed-rank test was used to test whether the values of RNA/DNA ratios of genera were significantly different in tumor *vs*. non-tumor tissue. (Prism7, GraphPad Software Inc, San Diego, CA).

The program PRIMER7 [40] with PERMANOVA+ [48] was used to test for differences in the microbial community structure among mucosal- rDNA and rRNA in dogs with tumors, between tumor tissue and adjacent non-tumor tissue, and between fecal rDNA in dogs with tumors and in control dogs. The weighted UniFrac distance matrix from mothur was used as input for permutation multivariate analysis of variance (PERMANOVA) with 10,000 permutations. Age and gender were implemented as covariates to evaluate whether these factors influenced the microbiota composition. We also used analysis of similarity (ANOSIM) within PRIMER 7 [40] on the weighted UniFrac distance matrix from mothur as well as the Bray-Curtis resemblance measure, using 10,000 permutations in order to test for significant differences in the fecal microbiota composition in dogs with tumor and control dogs. ANOSIM computes a p-value and an R value. In order to detect divergently expressed OTUs between the aforementioned clinical groups, we employed Linear Discriminant Effect Size (LEfSe) [49] analysis of the all-against-all type with no subclass. A p-value below 0.05 was considered statistically significant.

### Data accessibility

The 16S- rRNA and rDNA sequences have been deposited in the National Centre for Biotechnology Information (NCBI) Sequence Read Archive (SRA) with accession number SRP110343 under BioProject accession number: PRJNA391562.

## Results

### Animals

There were no significant differences in age, breed, weight and gender between dogs with tumors and control dogs (Mann Whitney U test, p>0.1).

### Sequencing analysis

A total of 5,464,587 sequences passed all quality control filters, with a mean of 99,356 sequences per sample (ranging from 5,955 to 410,693). The rarefaction curve of the alpha diversity metric “observed OTUs” reached a plateau in the majority of samples from the individual dogs, which indicates adequate sequencing depth (S3 File).

### Fecal microbiota in dogs with tumors and control dogs

**Fig 1:**
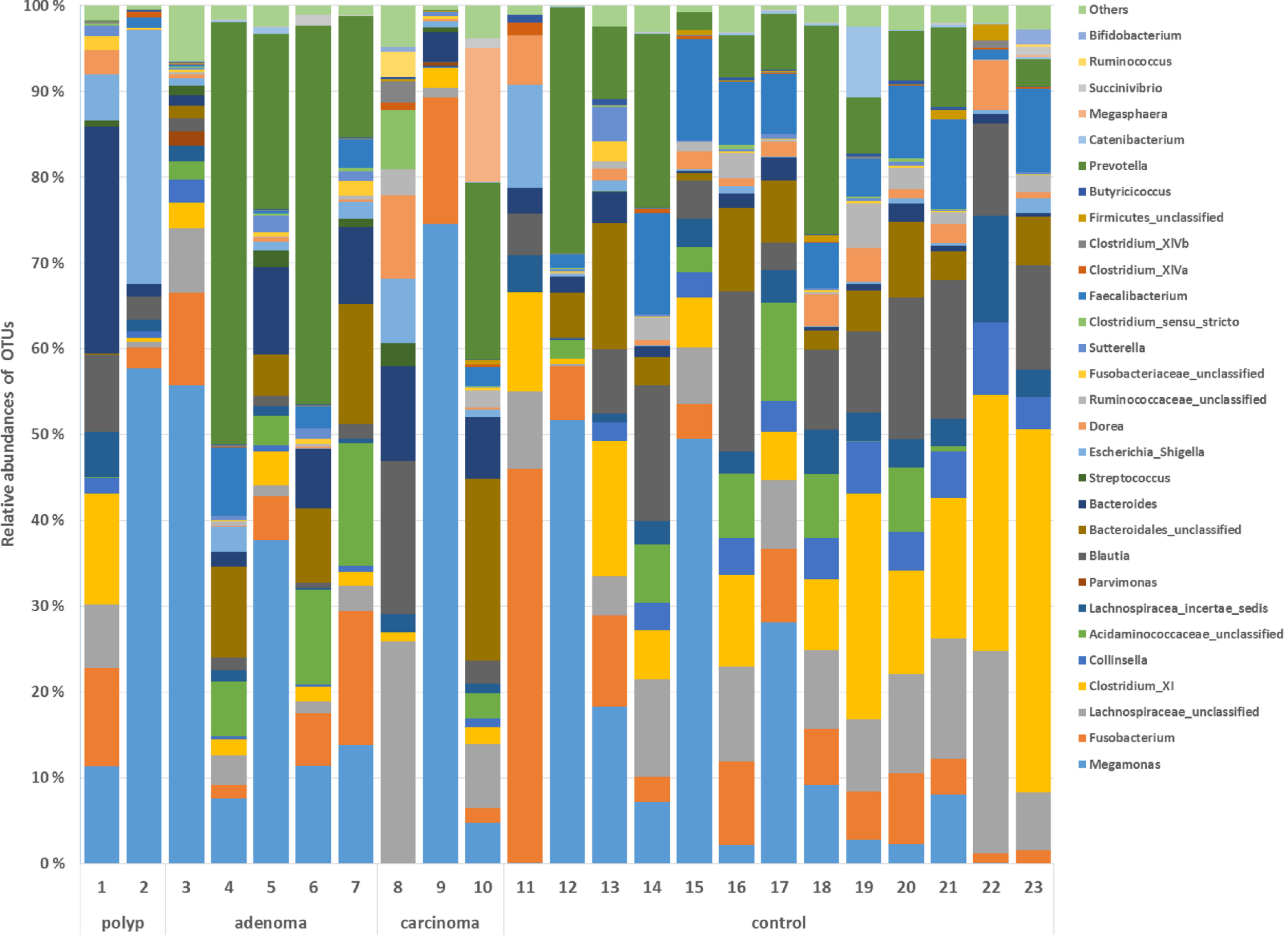
The relative abundance of OTUs at the genus level in fecal samples of control dogs and dogs with colorectal tumors (polyps, adenoma, carcinoma). The data are based on 16S rDNA and shows the 10 most abundant OTUs in each sample. Numbers at each bar base correspond to the “Dog id” in Table 1.

The most abundant phyla in tumor and control samples were *Firmicutes* (tumor mean ± st.dev, 56% ± 20; control mean ± st.dev, 68% ± 14%), *Bacteroidete*s (29% ± 23%; 16% ± 11%), *Proteobacteria* (7% ± 8%; 2% ± 3%) and *Actinobacteria* (1% ± 1%; 4% ± 3%). *Proteobacteria* were significantly overexpressed and *Actinobacteria* were significantly underexpressed in tumor samples (LEfSe, p < 0.05, LDA score >2). The most abundant genera in tumor and control samples were *Megamonas* (tumor mean ± st.dev, 27% ± 27%; control mean ± st.dev, 14% ± 18%), *Prevotella* (15% ± 19%; 9% ± 9%), *Bacteroides* (8% ± 8%; 2% ± 1%), *Fusobacterium* (7% ± 6%; 9% ± 12%), *Blautia* (4% ± 5%; 10% ± 6%), *Clostridium XI* (3% ± 4%; 15% ± 12%) and *Faecalibacterium* (2% ± 2%; 6% ±4%) (Fig 1). These genera have also been described in previous studies characterizing the canine fecal microbiota [50–53].

**Fig 2:**
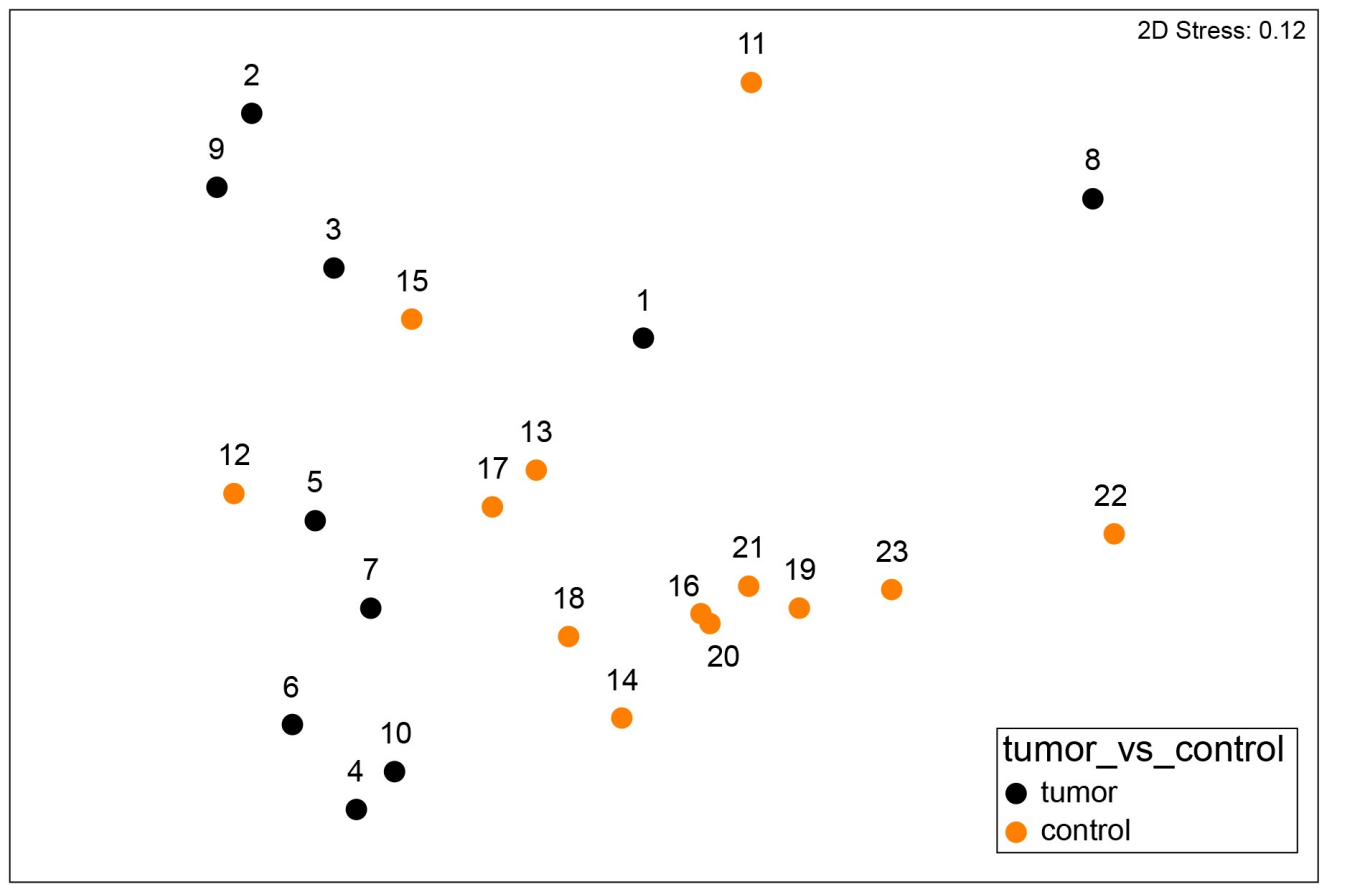
The bacterial community structure based on weighted UniFrac distance metric in fecal samples from dogs with tumors and control dogs. The nMDS plot shows the bacterial community structure in control dogs (orange, n = 13) and dogs with colorectal tumors (black, n = 10) based on the 16S rDNA data. Differences among these groups were significant (PERMANOVA, Pseudo-F = 3, p = 0.02 and ANOSIM, R Statistics = 0.27, p = 0.02).

**Fig 3:**
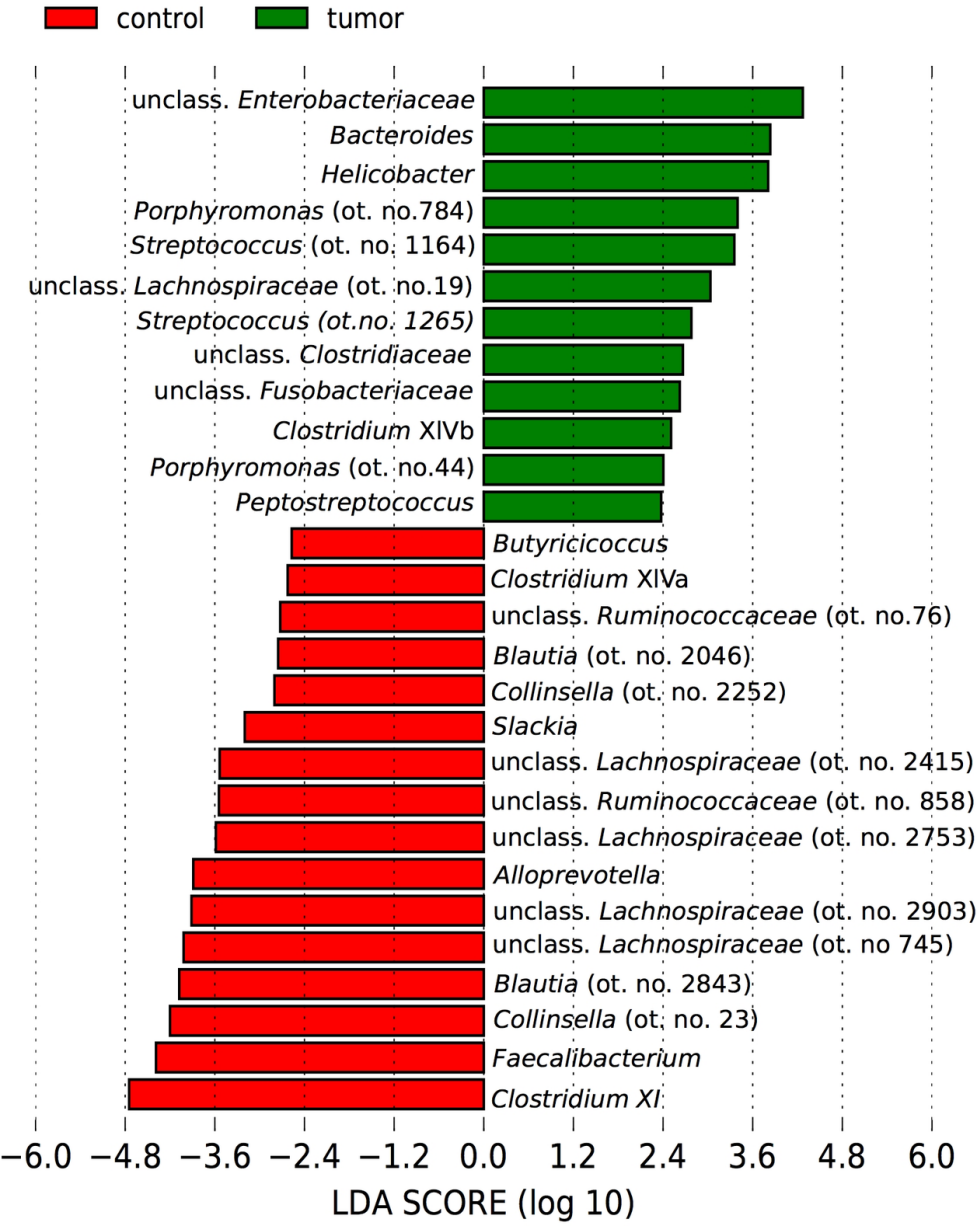
Differentially abundant bacterial taxa in fecal samples from dogs with tumors and control dogs. A bar plot showing the LDA score (effect size) of the oligotypes that were differentially abundant in fecal samples of control dogs (red, n = 13) and dogs with colorectal tumors (green, n = 10) as determined by Linear Discriminant Effect Size (LEfSe) analysis (α = 0.05, LDA score > 2.0). The number after the taxa name corresponds to the oligotype number (ot. no.).

The microbial community structure in fecal samples of dogs with tumors differed significantly from that of controls (PERMANOVA Pseudo-F = 3, p = 0.02) (Fig 2). ANOSIM also revealed significantly different communities between these groups based on the Weighted UniFrac measure (R Statistics = 0.27, p = 0.02) and the Bray-Curtis measure (R Statistics = 0.29, p = 0.01). The factors age and gender did not significantly influence the fecal microbiota in these dogs (PERMANOVA, age, Pseudo-F = 1.2, p = 0.3; gender, Pseudo-F = 0.7, p = 0.7). As revealed by Fig 2 and S4 File, samples from control dogs clustered more tightly compared with samples from dogs with tumors. Using LEfSe on the oligotypes obtained by MED analysis, a total of 28 oligotypes were differentially expressed between these two experimental groups (Fig 3). Tumor samples were characterized by oligotypes affiliated with *Enterobacteriaceae* (mean ± st.dev, 5% ± 9%) and several low abundance oligotypes (< 1% of the median values) including *Bacteroides*, *Helicobacter*, *Porphyromonas*, *Streptococcus*, *Peptostreptococcus* and *Fusobacteriaceae*. Control samples were characterized by oligotypes affiliated with *Clostridium* XI (14% ± 12%), *Faecalibacterium* (6% ± 5%), *Collinsella* (ot. no. 23, 4% ± 2%), unclassified *Lachnospiracea* (oligotype no.745, 3% ± 2%), *Blautia* (3% ± 3%), unclassified *Lachnospiraceae* (oligotype no. 2903, 2% ± 2%) and several low abundance oligotypes (<1% of the median values) including *Clostridium* XIVa, *Ruminococcaceae* and *Slackia*. The abundance of these genera are shown as boxplots in S5 File.

### Characterization of the mucosa-associated microbiota in dogs with colorectal tumors

**Fig 4:**
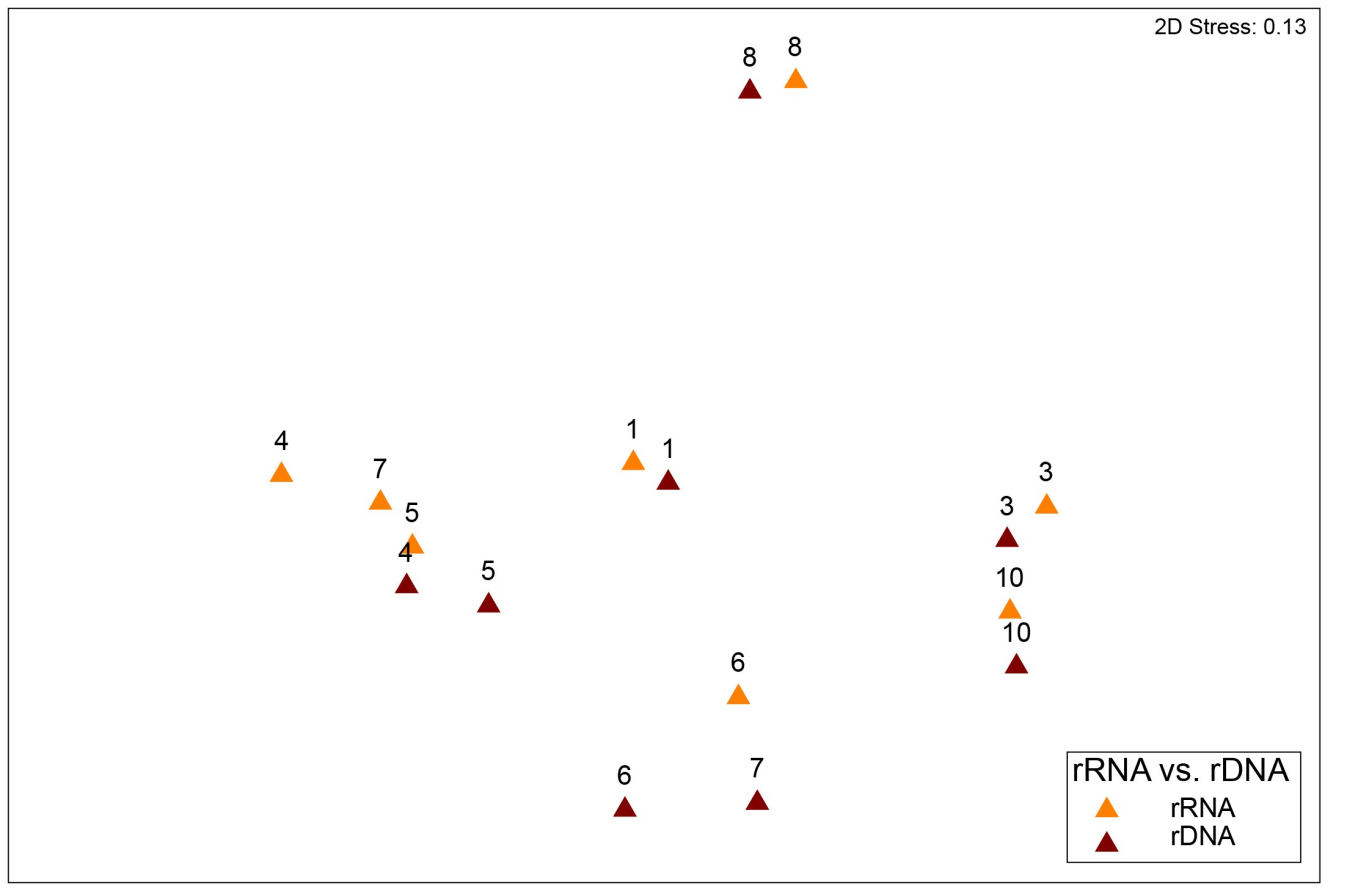
A non-metric multidimensional scaling (nMDS) plot based on the weighted UniFrac distance metric showing the bacterial community structure for paired mucosal samples at the 16S rDNA (brown) and 16S rRNA (orange) level from eight dogs with colorectal tumors. Numbers at each bar base correspond to the “Dog id” in Table 1. Differences between these groups were not significant (PERMANOVA p>0.1).

**Fig 5:**
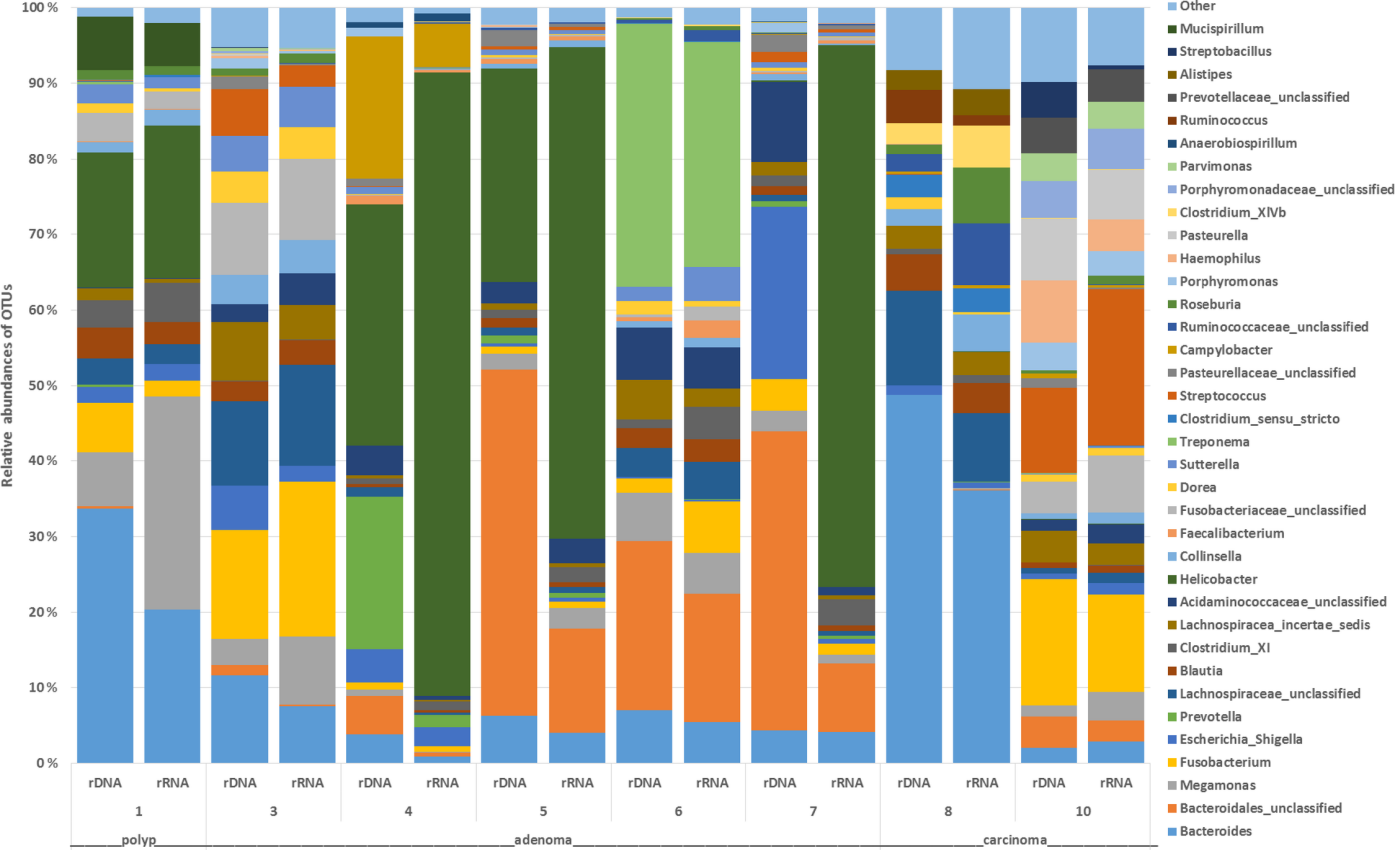
The relative abundance of OTUs at genus level in mucosal samples based on paired 16S rRNA and 16S rDNA data from 8 dogs with colorectal tumors (polyp, adenoma and carcinoma). Numbers at each bar base correspond to the “Dog id” in Table 1. The 10 most abundant OTUs in each sample are shown.

The microbial community structure in mucosal rDNA samples were not different from the rRNA samples (n = 8, PERMANOVA p>0.1) (Fig 4). Median values of the most abundant OTUs at genus level in tumor mucosal samples (at the rDNA level), were unclass. *Bacteroidales* (mean ± st.dev, 15% ± 19%), *Bacteroides* (15% ± 17%), *Helicobacter* (10% ± 14%), *Fusobacterium* (6% ± 6%), *Escherichia*/*Shigella* (5% ± 8%), *Treponema* (4% ± 12%), unclass. *Lachnospiraceae* (4% ± 5%), unclass. *Acidaminococcaceae* (4% ± 4%), *Lachnospiracea* incertae sedis (3% ± 2%), *Megomonas* (3% ± 3%), *Prevotella* (3% ± 7%) and *Campylobacter* (2% ± 7%) (Fig 5). For tumor mucosal samples at the rRNA level, *Helicobacter* (30% ± 37%), *Bacteroides* (10% ± 12%*)*, *Megamonas* (6% ± 9%), *Fusobacterium* (6% ± 7%), unclass. *Bacteroidales* (5% ± 7%), unclass. *Lachnospiraceae* (4% ± 5%), *Treponema* (4% ± 10%), *Streptococcus* (3% ± 7%), unclass. *Fusobacteriaceae* (3% ± 4%), *Clostridium* XI (2% ± 2%), unclass. *Acidaminococcaceae* (2% ± 2%), *Blautia* (2% ± 1%), *Collinsella* (2% ± 2%), *Lachnospiracea* incertae sedis (2% ± 2%) and *Sutterella* (2% ± 2%) were most abundant (Fig 5). No differentially expressed OTUs were detected between rRNA and rDNA samples (LEfSe).

**Fig 6:**
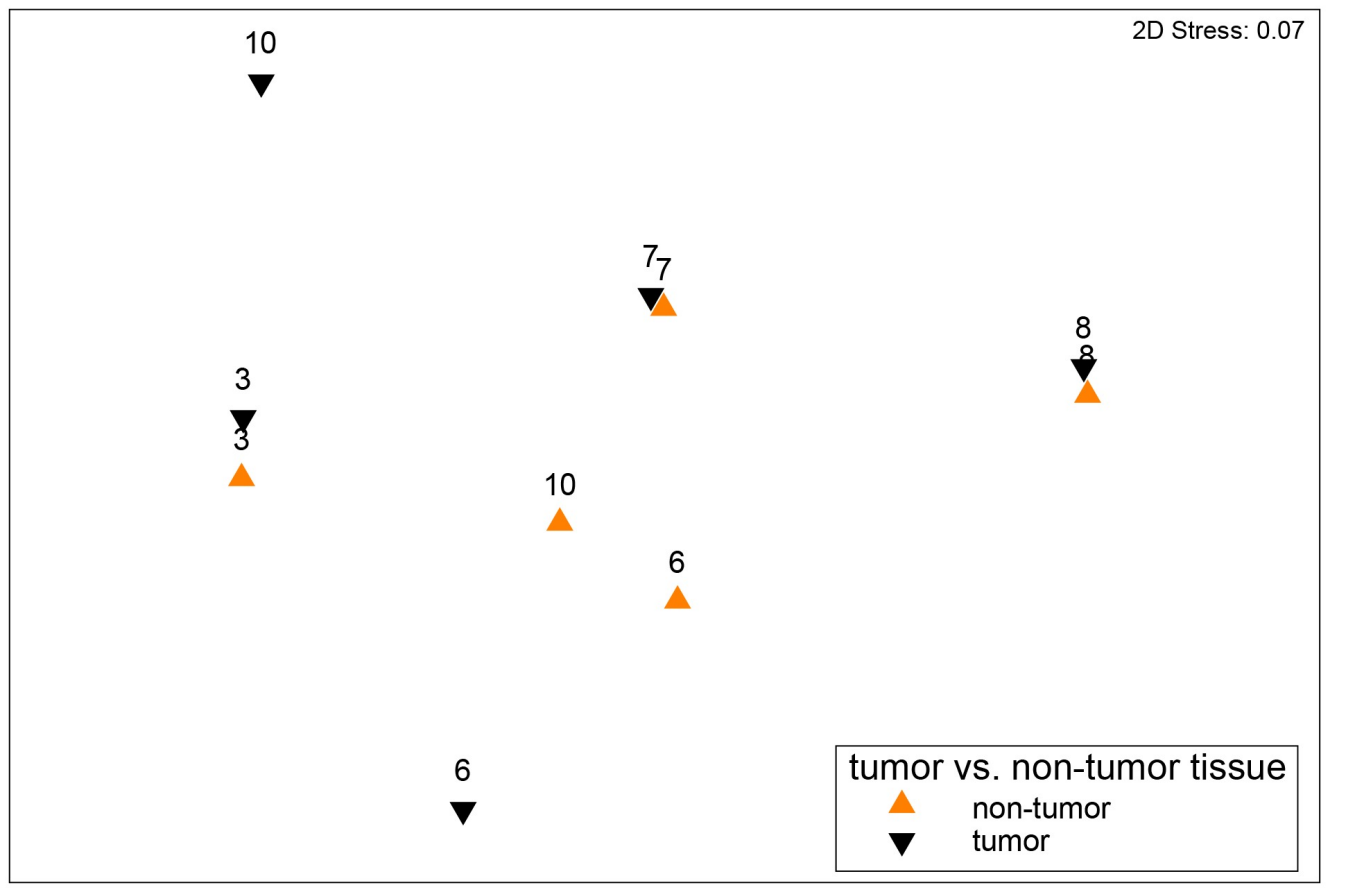
A non-metric multidimensional scaling (nMDS) plot based on the weighted UniFrac distance metric showing the microbial community structure based on tumor (black) and adjacent non-tumor tissue (orange) from five dogs with colorectal tumors. The data are based on the 16S rDNA data. Labels adjacent to data points correspond to the “Dog id” in Table 1. Differences between these groups were not significant (PERMANOVA p>0.1).

The microbial community structure in mucosal tumor tissue was not different from that of adjacent non-tumor tissue based on the rRNA and the rDNA data (n = 5, PERMANOVA, p>0.1) (Fig 6).

**Fig 7:**
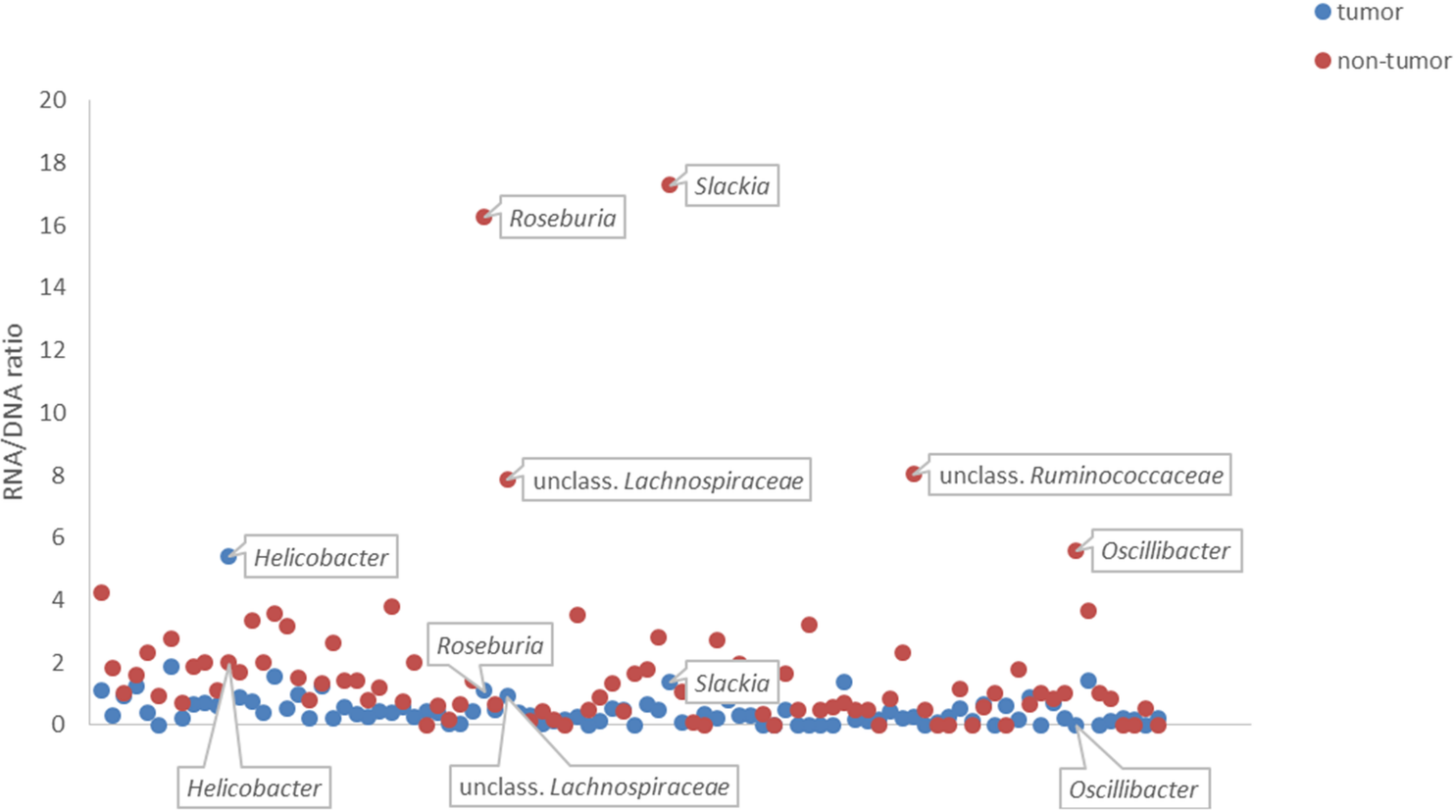
A scatterplot showing the ratio of live, potentially active bacteria (RNA/DNA) in tumor *vs*. non-tumor tissue.

The ratio of live, potentially active bacteria appeared to be higher in non-tumor tissue *vs*. tumor tissue (Fig 7). The genera that contributed most to these differences were unclass. *Lachnospiraceae*, *Oscillibacter*, *Roseburia*, unclass. *Ruminococcaceae* and *Slackia*, which appeared to be more active in non-tumor tissue compared with tumor tissue. However, none of these results were statistically significant (Wilcoxon signed-rank test, p>0.1). Stacked bar plots of the ten most abundant OTUs at genus level in tumor and non-tumor tissue are found in S6 File.

### The alpha diversity

No significant differences were detected in evenness and richness between the mucosal rDNA and mucosal rRNA samples, between tumor tissue and adjacent non-tumor tissue, or between fecal samples at the rDNA-level from dogs with tumors and control dogs (Wilcoxon matched-pairs signed rank test for paired data and Mann-Whitney U test for unpaired data p>0.1) (S7 File).

## Discussion

The intestinal microbiota, dominated by bacteria, is believed to have a major influence on host health and wellbeing [54]. Dysbiosis, an unhealthy disruption in the intestinal bacterial community, has been described in humans with early and late stages of colorectal cancer [55, 56]. Although colorectal cancer in dogs is rare, and therefore less characterized as compared to humans, studies have suggested similarities in the etiopathogenesis in these species [57, 58]. To our knowledge, this is the first study to give detailed insight into both the fecal- and mucosa-associated microbiota in dogs diagnosed with colorectal polyps, adenomas and carcinomas.

We observed a significantly different fecal microbiota profile in dogs with tumors as compared with that of controls, where *Enterobacteriaceae*, *Bacteroides*, *Helicobacter*, *Porphyromonas*, *Streptococcus* and *Fusobacteriaceae* were overrepresented in the dogs with tumors. All of these, except *Enterobacteriaceae*, were present in low abundance (<1% of the median relative abundances in dogs with tumors). Low-abundant bacteria may have clinical relevance if they have pathogenic potential (e.g. increased adherence/invasiveness to the mucosal surface, toxin productions etc.) [59]. Interestingly, these bacteria have been identified as potential contributors to human colorectal tumorigenesis [10, 16, 60]. In humans, *Helicobacter pylori* is linked to gastric cancer [61]. It may also participate in the pathogenesis of human colorectal cancer, although this association is more uncertain [11]. In 4 out of 8 dogs, *Helicobacteriaceae* were an abundant and potentially active component (based on the rRNA sequence data) of the mucosa- associated microbiota (Fig 5). Whether *Helicobacter* spp. play a role in the development of gastric diseases in dogs has not yet been established, and needs further investigation [62]. The relevance of this bacteria in canine intestinal disorders is also unclear. A recent study based on HTS observed that unclass. *Helicobacteriaceae* was enriched in colonic mucosal microbiota of client-owned dogs with food-responsive enteropathies [63]. However, a study of laboratory dogs showed higher abundance of *Helicobacter* spp. in healthy colorectal tissue *vs*. colorectal cancerous tissue (adenocarcinoma, n = 9; lymphosarcoma, n = 3), dogs with IBD (n = 19) and dogs with granulomatous colitis (n = 6), based on fluorescent in situ hybridization (FISH) [22]. The latter study also observed an increased number of mucosa-adherent *Enterobacteriaceae*, including *Escherichia coli* and *Bacteroides* spp. in tumor samples as compared with healthy control samples [22]. It should be noted that laboratory dogs may not necessarily represent the pet dog population. For example *Helicobacter* spp. was more abundant in the gastric microbiota of laboratory and shelter dogs as compared with pet dogs [64]. We observed *Helicobacteriaceae*, *Enterobacteriaceae* and *Bacteroides* spp. in mucosal tumor tissue, but could not determine whether their presence was unique to tumor samples due to the lack of mucosal samples from control dogs. Future work should entail prospective case-control studies whereby control samples are collected with the owner's permission from dogs euthanized for non-gastrointestinal disorders during necropsy.

Overrepresentation of oral-originating bacteria, including *Fusobacterium*, *Peptostreptococcus* and *Porphyromonas* in fecal microbiota, has been observed in humans with colorectal adenoma and carcinoma [17, 65–68]. These bacteria are also part of the canine oral microbiota [69] and were in the present study, found to be overexpressed in the fecal microbiota in dogs with tumors. It is hypothesized that colonization of opportunistic pathogenic bacteria not normally present in the colonic microenvironment might be a result of alterations [17] such as changes in nutrients (e.g. amino acids, fatty acids, glucose, and pyruvate) [70], or inflammation [71]. Colorectal tumorigenesis is therefore thought to be associated with a shift in the entire community of bacteria [17].

The fecal microbiota in our dogs with tumors was characterized by an under expression of *Ruminococcaceae*, *Faecalibacterium*, *Slackia and Clostridium* XIVa. These bacteria are efficient producers of the anti-inflammatory and anti-carcinogenic metabolite butyrate [72]. A similar reduction of efficient butyrate producers, in particular *Clostridium* XIVa, have been identified in human patients with colorectal adenoma and carcinoma [68, 73–75]. Whether the reduction of potentially health-promoting bacteria has consequences for tumor development in dogs, or is rather a result of tumor development, calls for further investigation.

Studies in humans have reported differences in the abundance of bacterial taxa between mucosal samples from tumorous and adjacent non-tumorous tissue [28, 56]. However, in a study of humans with colorectal carcinoma, non-adjacent tumor tissue was collected 10–30 cm distal as well as proximal to the tumor, and no significant differences in microbiota structure were observed between these locations [27]. Our results showed that the mucosa-associated microbiota composition was not restricted to tumor tissue, but was also present in adjacent non-tumor tissue. Although it was not significant, the proportion of live, potentially active bacteria appeared to be higher in non-tumor tissue compared with tumor tissue and included the genera *Slackia*, *Roseburia*, unclass. *Ruminococcaeceae* and unclass *Lachnospiraceae* and *Oscillibacter*. The lower proportion of live and potentially active members of *Ruminococcaeceae* and *Lachnospiraceae* in tumor tissue may result in lower production of butyrate and reduced defense mechanisms against tumor development [76]. *Oscillibacter* has been found in the human fecal microbiota [77] and in the kitten fecal microbiota [78]. It was more abundant in the healthy human fecal microbiota as compared with patients with Crohn's disease [77]. Whether this genus impacts canine intestinal health, is currently unknown. Methods such as FISH or qPCR could be used to determine whether there are low-abundance, pathogenic bacteria not detected with methods used in the present study that are associated with tumor tissue [12, 79–81]. Importantly, since samples were collected in dogs where tumors had already developed, it is impossible to determine whether the fecal- and mucosa-associated microbiota in these dogs was present prior to (rather than as a result of) the tumor development. It would be unethical to collect mucosal samples through colonoscopy in dogs on a regular basis, in order to detect potential changes in the intestinal microbiota along the colorectal tumorigenesis. It could however be achieved with fecal samples, as these are collected non-invasively. However, such longitudinal studies would be expensive and long-term, particularly since colorectal cancer is rarely diagnosed in dogs [4, 82, 83].

In the UK, the age-standardized incidence rate of colonic tumors was 8/100,000 dogs per year from 1997 to 1998 [84]. The rarity of this disorder thus limited the number of dogs included in this study. The dogs, including the healthy controls, represent a heterogeneous population consisting of different breeds, ages and genders and were raised in different environments and under different diet regimes. All of these factors could influence the composition of the mucosal and fecal microbiota. We found no significant difference in the fecal microbiota composition of dogs due to age or gender. Our previous study [30], as well as those of others [85, 86], have found that large shifts in the macronutrient composition is necessary in order to change the fecal microbial communities. Dogs in our study received different types of dry food, but the composition of macronutrients in these diets was not as extreme as in the aforementioned studies. Worth noting is that diet may have confounded our results, as 10 of 13 control dogs received similar dry food for two weeks prior to sample collection, whereas dogs with tumors were fed various types of dry food. This may explain why the interindividual variation in the fecal microbiota composition among control dogs was lower as compared with dogs with tumors. Previous studies have revealed a larger interindividual variation among IBD dogs as compared with control dogs [19, 20]. In those prior studies, all dogs received various types of diets and thus diet was not the principal cause of their results. The similarities within the fecal microbiota composition in IBD dogs and the dogs with colorectal tumors (increased *Proteobacteria* and reduced *Firmicutes*) in the present study may indicate a common underlying cause, for example inflammation. Comparing the intestinal microbiota in dogs with various chronic enteropathies to determine whether there is a distinct microbial signature associated with specific disorders would be valuable. In this context, it would be important to consider diet as a confounding variable and feed all dogs (sick and control dogs) a similar diet. However, convincing owners of dogs with tumors to feed their dog a specific diet solely for the benefit of research could prove difficult, as the dogs may prefer some diets to others, or their skin/fur quality and gastrointestinal function may improve on particular diets. Moreover, the withholding of food and the bowel cleansing treatment prior to colonoscopy and surgery influence the mucosa-associated microbiota [63, 87]. However, these factors are difficult to avoid in clinical scenarios. To avoid the influence of antibiotics on the intestinal microbiota, samples from dogs with tumors having received antibiotics within last the three months prior to sample collection were excluded. Antibiotics are sometimes used during the clinical workup of dogs with chronic enteropathies [88], and excluding dogs treated with antibiotics further decreased the number of dogs in this study. This was also the reason why we could not apply a six month cut-off for including dogs with tumors, although control dogs had not received antibiotics for at least six months prior to sample collection. Although previous studies in dogs have showed that the fecal microbiota in dogs was restored in most dogs within 14 days after cessation of antibiotics, some bacterial taxa failed to recover [89, 90]. In a human study it was also observed that some bacterial taxa failed to recover within a period of six months after treatment with antibiotics [91]. Although the time-frame is important, factors that determine whether antibiotics cause permanent shifts in the microbiota are also whether the antibiotics are broad- or narrow-spectrum, and whether the treatment is given during juvenile or adult stages during life development [92]. Therefore we cannot rule out that antibiotic treatment prior to three (tumor dogs) or six months (control dogs) had not caused permanent changes of the intestinal microbiota in some of our dogs.

Altogether, our study generates hypotheses which can inform future studies that should include breed- and age-matched case-controls in order to evaluate the impact of the intestinal microbiota on the etiopathogenesis of canine colorectal epithelial tumors. In order to accomplish this, collaborations between clinicians working at large hospitals in several countries and collecting samples over several years would be required.

## Conclusions

The fecal microbiota composition in dogs with colorectal epithelial tumors was different from that of control dogs and consisted of low-abundance but potentially pathogenic bacteria as well a reduction of possible health-promoting bacteria within *Clostridiales*. The mucosa-associated microbiota composition was not restricted to tumor tissue but was also present in adjacent non-tumor tissue, indicating that the microbiota was unlikely to have resulted from localized tumor changes, such as inflammation and ulcerations. Our results provide knowledge which might be helpful for future research into the etiopathogenesis of canine colorectal tumorigenesis as well for the development of bacterial biomarkers to screen for the disease.

## Supporting information

S1 File Diets given to control dogs prior to being fed Felleskjøpet Labb Adult dry food for two weeks. (XLSX)

S2 File Indexed primers. The nucleotide sequences of the indexed primers used for PCR of the V4 region of the 16S rDNA- and rRNA.
(XLSX)

S3 File Rarefaction curve of V4 16S rDNA- and cDNA sequences calculated at 3% OTU dissimilarity showing observed OTUs per individual dog. The analysis was performed on a randomly selected subset of 5500 sequences per sample. Average number of observed species and corresponding error bars representing standard deviation are shown for each dog.
(DOCX)

S4 File **The bacterial community structure based on Bray Curtis (A) and Jaccard distances (B) in fecal samples of dogs with tumors and control dogs.** The nMDS plots show the bacterial communities in fecal samples from control dogs (orange, n = 13) and dogs with colorectal tumors (black, n = 10). ANOSIM on the Bray-Curtis measure revealed that these communities were significantly different (R Statistics = 0.29, p = 0.01).
(PDF)

S5 File Box plots of the divergently expressed genera in fecal microbiota in dogs with tumors *vs* control dogs. The data are based on 16S rDNA and shows median values and interquartile ranges of the different oligotypes.
(PDF)

S6 File The relative abundance of OTUs at genus level in tumor and adjacent non-tumor samples in five dogs with colorectal tumors (adenoma and carcinoma). The data are based on the 16S rDNA (A), and the 16S rRNA (B). Numbers at each bar base correspond to the “Dog id” in Table 1.
(TIF)

S7 File Diversity analysis of the 16S rDNA- and rRNA sequence data. Alpha diversity parameters (median values, with min. and max. ranges) evaluated in mucosal samples at the rRNA and rDNA level from tumor tissue and from adjacent non-tumor tissue, as well as from fecal samples at the rDNA level from dogs with tumors and from control dogs. No significant differences were found between these groups (p>0.1) (evaluated by Wilcoxon matched-pairs signed rank test for paired samples and by Mann Whitney U test for unpaired samples).
(DOCX)

## Abbreviations

Sobs: number of observed OTUs
NpShannon: non-parametric Shannon-diversity index
InvSimpson: inverse Simpson diversity index
