# Supplementary figures and images for: Characterization of the fecal and mucosa-associated microbiota in dogs with colorectal epithelial tumors
Source: PLoS One. 2018 May 31;13(5):e0198342. doi: 10.1371/journal.pone.0198342 (PMC5979030; doi:10.1371/journal.pone.0198342)

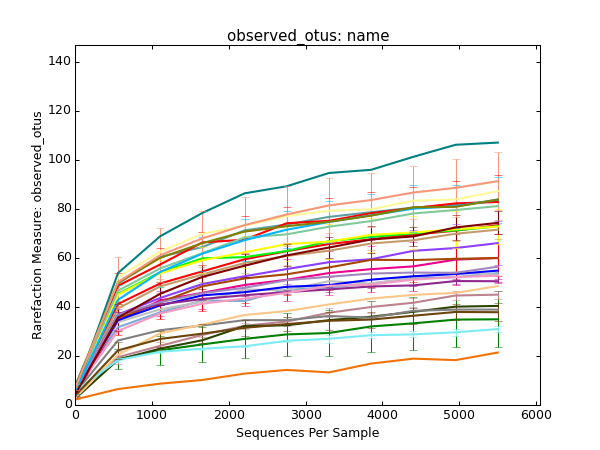

Supplement: S3 File — The analysis was performed on a randomly selected subset of 5500 sequences per sample. Average number of observed species and corresponding error bars representing standard deviation are shown for each dog. (DOCX) [file pone.0198342.s003.docx]

Bray-Curtis NMDS tumor vs. control

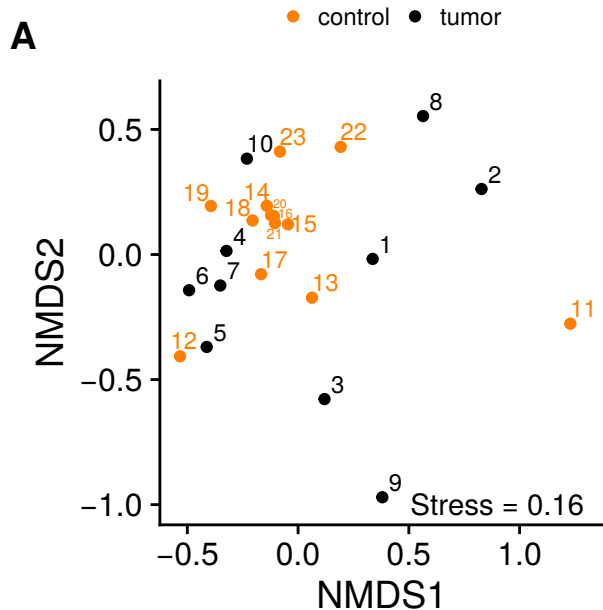

Jaccard NMDS tumor vs. control

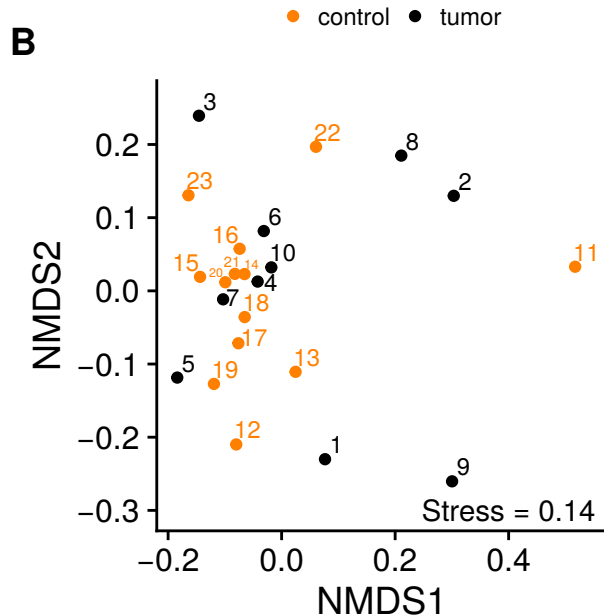

Supplement: S4 File — The bacterial community structure based on Bray Curtis (A) and Jaccard distances (B) in fecal samples of dogs with tumors and control dogs. The nMDS plots show the bacterial communities in fecal samples from control dogs (orange, n = 13) and dogs with colorectal tumors (black, n = 10). ANOSIM on the Bray-Curtis measure revealed that these communities were significantly different (R Statistics = 0.29, p = 0.01). (PDF) [file pone.0198342.s004.pdf]

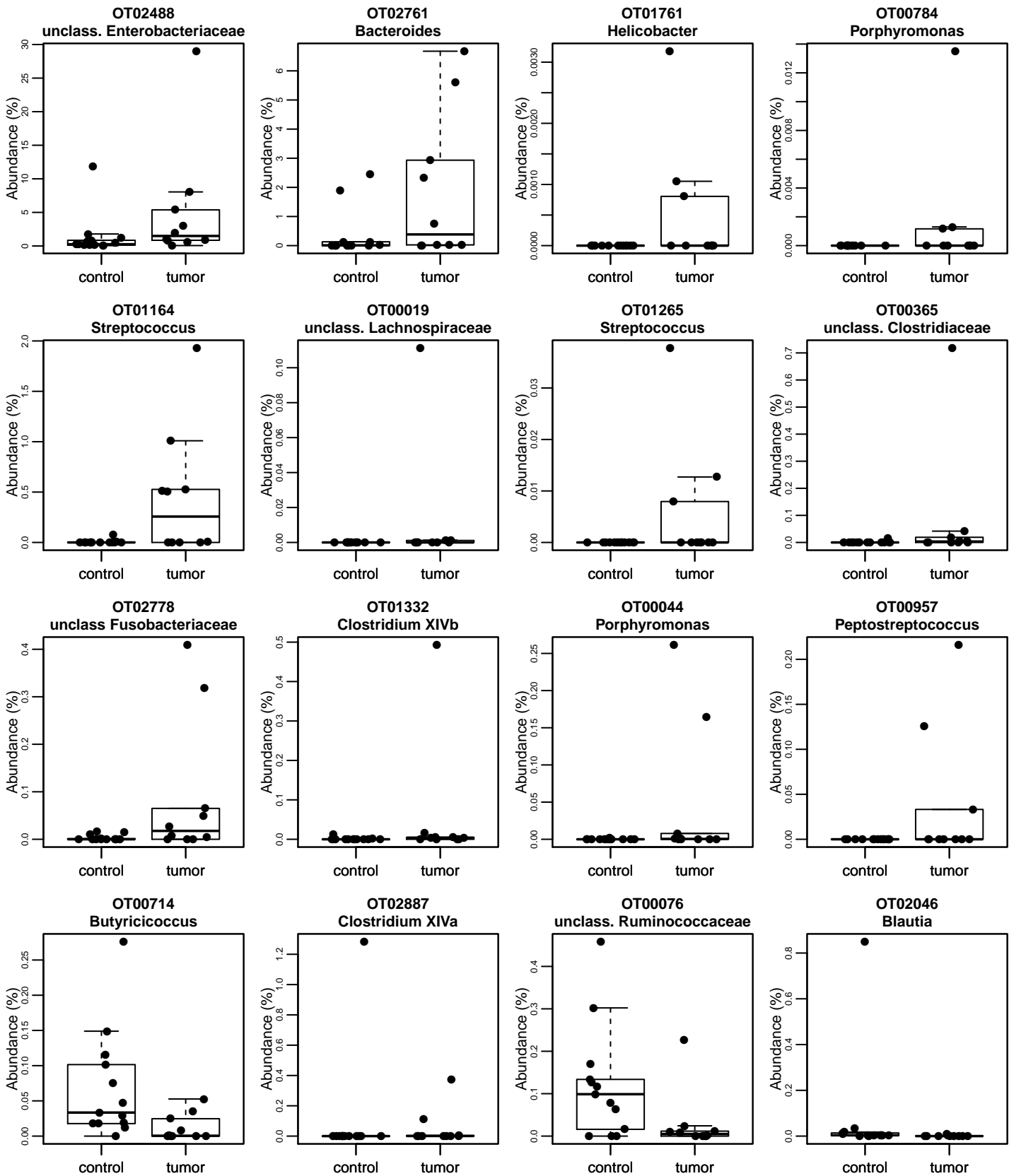

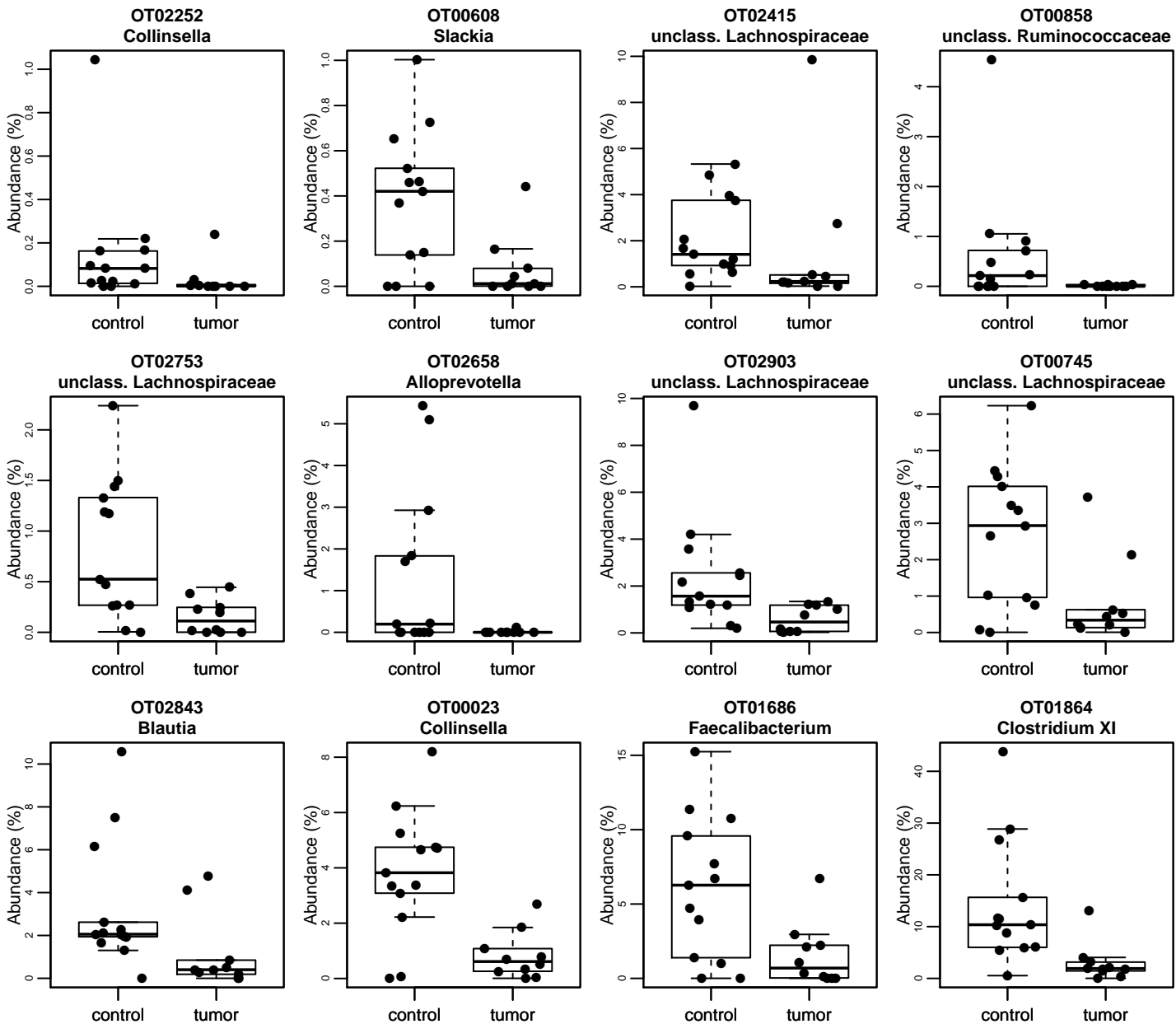

Supplement: S5 File — The data are based on 16S rDNA and shows median values and interquartile ranges of the different oligotypes. (PDF) [file pone.0198342.s005.pdf]

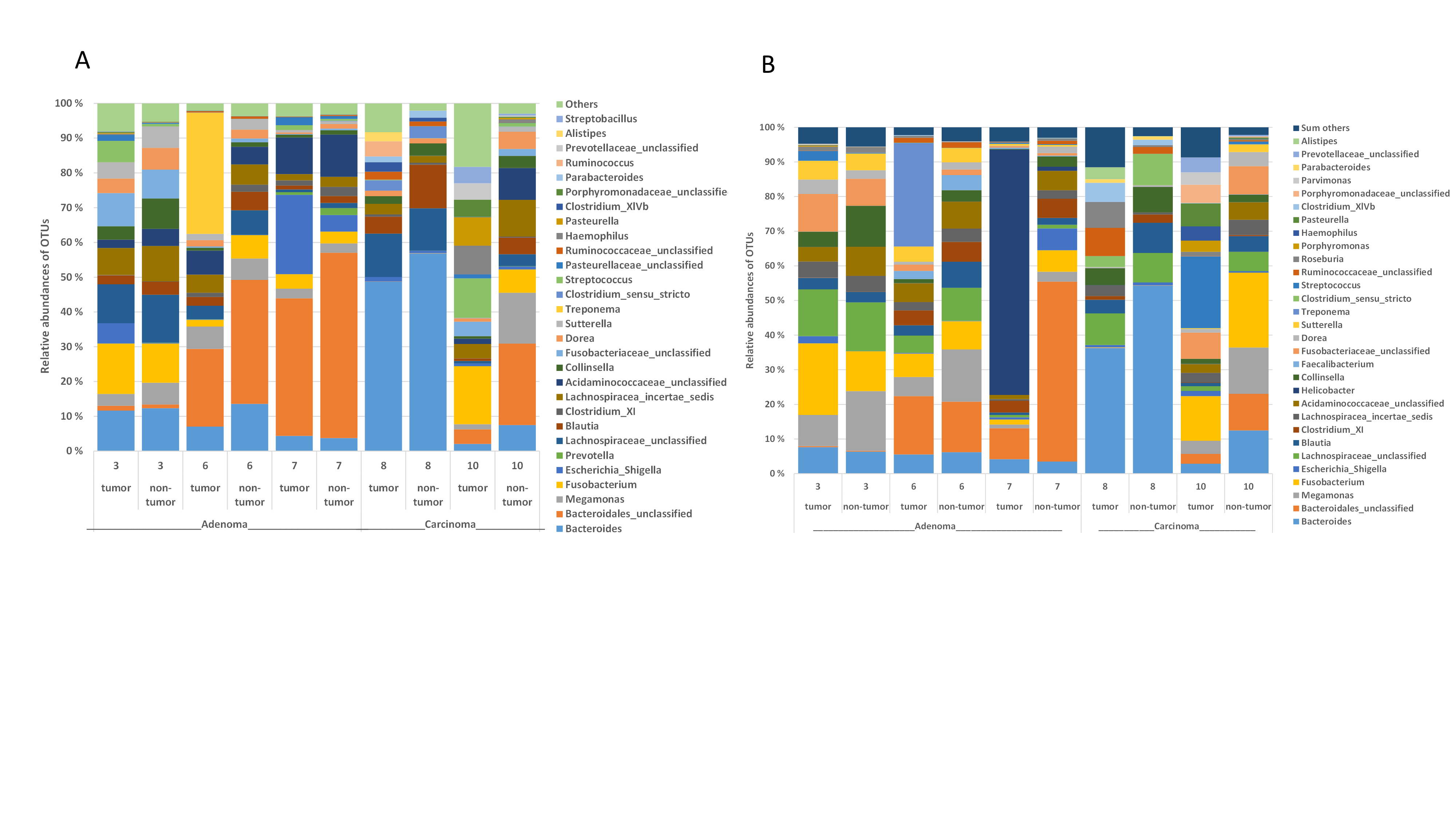

Supplement: S6 File — The data are based on the 16S rDNA (A), and the 16S rRNA (B). Numbers at each bar base correspond to the “Dog id” in Table 1. (TIF) [file pone.0198342.s006.tif]
