# Supplementary material for: Characterization of the fecal and mucosa-associated microbiota in dogs with colorectal epithelial tumors
Source: PLoS One. 2018 May 31;13(5):e0198342. doi: 10.1371/journal.pone.0198342 (PMC5979030; doi:10.1371/journal.pone.0198342)
Supplement: S7 File — Alpha diversity parameters (median values, with min. and max. ranges) evaluated in mucosal samples at the rRNA and rDNA level from tumor tissue and from adjacent non-tumor tissue, as well as from fecal samples at the rDNA level from dogs with tumors and from control dogs. No significant differences were found between these groups (p>0.1) (evaluated by Wilcoxon matched-pairs signed rank test for paired samples and by Mann Whitney U test for unpaired samples). (DOCX) [file pone.0198342.s007.docx]

**S7 file**

|  | Mucosal rRNA in tumor tissue  (n=5) | Mucosal rDNA in tumor tissue  (n=5) | Mucosal rRNA in adjacent non-tumor tissue  (n=8) | Mucosal rDNA in adjacent non-tumor tissue  (n=8) | Fecal rDNA in dogs with tumors  (n=10) | Fecal rDNA in control dogs  (n=13) |
| --- | --- | --- | --- | --- | --- | --- |
| Sobs | 74(61-96) | 71(65-105) | 73(64-96) | 79(64-84) | 71(21-111) | 55(31-106) |
| NpShannon | 2.6(1.1-3.3) | 2.5 (2-3.3) | 2.8(2.5-3.1) | 2.7(2.3-3) | 2.3 (1-3.3) | 2.8(1.5-3.3) |
| InvSimpson | 7.1(1.5-13.6) | 6.5(3.4-17.3) | 11.7(4.5-13.8) | 5.6(3.417.3) | 4.7(1.718.4) | 9.5(2.8-18.3) |
